# Supplementary material for: Acceptability and feasibility of integrating female genital schistosomiasis and sexual and reproductive health interventions in Kenya: A demonstration study
Source: PLOS Glob Public Health. 2025 Sep 17;5(9):e0004938. doi: 10.1371/journal.pgph.0004938 (PMC12443286; doi:10.1371/journal.pgph.0004938)
Supplement: S1 Text — (DOCX) [file pgph.0004938.s001.docx]

# Supplementary File 1

## Female Genital Schistosomiasis (FGS) Risk Screening Checklist (1)

**Part A: Environmental Risk Assessment**

1. Since you were a child, have you done the following in freshwater/stagnant water, for instance rivers, ponds, small lakes?
   - Fetching water
   - Washing clothes
   - Bathing
   - Swimming
   - Playing
   - Fishing
   - Farming
2. What is the distance from your home compound to your nearest natural water source(s) e.g., lake, river, stream, dam?

<5km, 5-15km, >15km?

1. What other sources are available locally, e.g., well, borehole, water truck

**Part B: Checking for FGS Signs and Symptoms**

| Question | Circle your response | | |
| --- | --- | --- | --- |
| Do you have genital itching or burning? If yes, how severe? | Mild | Moderate | Severe |
| Do you have pain in your area – below your tummy? If yes, how severe? | Mild | Moderate | Severe |
| Do you have vaginal discharge? If yes, how heavy is the discharge? | Mild | Moderate | Severe |
| Do you find it difficult to control your pee? (involuntary urination). If yes, does this happen often? | Rarely | Occasionally | All the time |
| Do you bleed while passing urine? | Rarely | Occasionally | All the time |

| Do you have pain during sex? If yes, how often?* | Yes | N/A or No | Occasionally | All the time |
| --- | --- | --- | --- | --- |
| Do you have spotting/bleeding during/after sex?* | Yes | N/A or No | Occasionally | All the time |
| Do you have irregular periods? | Yes | No | Occasionally | All the time |
| Have you had previous treatment for STIs, infertility, cervical cancer screening? | Yes |  | Occasionally | All the time |

# Reference

1. Countdown consortium. Countdown FGS Training Guide for healthcare workers, Liberia – Proof 3 [Internet]. Liverpool: Liverpool School of Tropical Medicine; 2021 May [cited 2025 Jun 10]. Available from: https://countdown.lstmed.ac.uk/sites/default/files/centre/Countdown%20FGS%20Training%20Guide%20Liberia%20-%20Proof%203-%20watermarked.pdf
