# Supplementary material for: Acceptability and feasibility of integrating female genital schistosomiasis and sexual and reproductive health interventions in Kenya: A demonstration study
Source: PLOS Glob Public Health. 2025 Sep 17;5(9):e0004938. doi: 10.1371/journal.pgph.0004938 (PMC12443286; doi:10.1371/journal.pgph.0004938)
Supplement: S2 Text — (DOCX) [file pgph.0004938.s002.docx]

**Supplementary Information 2**

**Data Collection Tools**

Contents

[1. IN-DEPTH INTERVIEW (IDI) GUIDE FOR HEALTHCARE WORKERS (HCWs) 2](#_Toc199524862)

[2. IN-DEPTH INTERVIEW (IDI) GUIDE FOR CHPs/CHAs/PEs 4](#_Toc199524863)

[3. IN-DEPTH INTERVIEW (IDI) GUIDE FOR COMMUNITY GATE KEEPERS 6](#_Toc199524864)

[4. IN-DEPTH INTERVIEW (IDI) GUIDE FOR WOMEN/GIRLS 9](#_Toc199524865)

[5. FOCUS GROUP DISCUSSION (FGD) GUIDE FOR CHPs/CHAs/PEs 12](#_Toc199524866)

[6. FOCUS GROUP DISCUSSION (FGD) GUIDE FOR WOMEN/GIRLS 15](#_Toc199524867)

[7. FOCUS GROUP DISCUSSION (FGD) GUIDE FOR COMMUNITY GATE KEEPERS 18](#_Toc199524868)

[8. KEY INFORMANT INTERVIEW (KII) GUIDE FOR: HEALTH MANAGERS 21](#_Toc199524869)

[9. Survey tool for acceptability of integrating FGS in SRH based on the Theoretical framework for Acceptability for female clients (15-49 years) 23](#_Toc199524870)

[10. Survey tool for acceptability of integrating FGS in SRH based on the Theoretical framework for Acceptability for Health Workers 25](#_Toc199524871)

# IN-DEPTH INTERVIEW (IDI) GUIDE FOR HEALTHCARE WORKERS (HCWs)

| *Select type of participant* | |
| --- | --- |
| *Healthcare worker – specify cadre (1)* | *Health manager – specify position (2)* |

| Date of IDI: | __ __ / __ __ / __ __ __ __ (dd-mmm-yyyy) |
| --- | --- |
| Site Number: | ________________ / _________________ |
| Research Assistant’s Name: | ___________________________________ |
| Type of Participant (*Use code above* – *specify cadre*) | ______ -________ |
| IDI number: | _______________________________ |
| Start time (in 24 hour): | ________-Hours |
| End time (in 24 hour): | _______ - Hours |
| Duration of the interview: | ____: ____ (Minutes: Hours) |

**NB:** *Sections in* ***Italics*** *should not be read to participant. These are instructions to study staff.*

***Reminder:*** *After each question is asked, participants should be given plenty of time to respond. Be comfortable with silence, do not fill the silence with probes. Probes should be used to gather more information about the participant’s response, especially in the case of quiet participants. Probes are not required to be read. Probes should only be used when needed. Do not use more than one probe at once.*

1. ***Introduction***
2. Please tell me about your designation at this health facility
3. What are your primary duties?

**Now, I would like to ask you some general questions about the topic of our discussion:**

1. In your own words, what is schistosomiasis?

*Probe: How is it called by patients from this community?*

1. What is female genital schistosomiasis (FGS)?
2. What SRH services are offered in this facility?

*Probe the kind of SRH services (diagnosis, counselling, treatment)*

1. Where are the SRH services offered?
2. How involved are you in the delivery of SRH services?
3. What Schistosomiasis services are provided in this facility?

*Probe the kind of schistosomiasis services (diagnosis, counselling, treatment)*

1. How are you involved in the delivery of schistosomiasis services?
2. Who are the main clients that seek Schistosomiasis services?
3. Do you know how to diagnose female genital schistosomiasis (FGS)?
4. What services for addressing FGS are provided in this facility?
5. **Acceptability of integrating FGS and SRH**
6. How would you integrate FGS and SRH services in this facility?
7. In your view, what would be the implications of integrating FGS and SRH services?
   - *Probe for each reason provided*
8. How confident are you about providing integrated FGS and SRH services?
   - *Please tell us why or why not.*
9. What would need to be changed in this facility to enable the integration of FGS and SRH services?
   - *Probe each point shared*
10. Overall, how acceptable do you think it will be to integrate FGS and SRH services in this facility? How do you think that staff will accept it? How do you think that the clients will accept it?
    - *Probe the reasons*
11. **Feasibility of integrating FGS and SRH**
12. What kind of support do you think you would need for FGS and SRH to be integrated in this facility?
    - Probe:
      1. *Service delivery eg space, commodities etc?*
      2. *Equipment eg specific equipment?*
      3. *Human resources eg a specific cadre?*
      4. *Finance eg procurement financial support?*
      5. *Health information/Registers eg how is FGS recorded, which tools?*
13. Based on your experience in this facility, what are the potential barriers for integrating FGS and SRH services?
    - *Probe: Workload, privacy and confidentiality, medical supplies, staffing, training, IEC materials, registers*
14. What would be the enablers for integrating FGS and SRH in this facility?

Thank you so much for your time today. Do you have any additional thoughts or questions you would like to share with me?

**End Time**: ______ Hours

# IN-DEPTH INTERVIEW (IDI) GUIDE FOR CHPs/CHAs/PEs

| ***Select Participant by type*** | | |
| --- | --- | --- |
| *Community Health Promoters (CHPs) (1)* | *Community Health Assistants (CHAs) (2)* | *Peer Educators (PE) (3)* |

| Date of IDI: | __ __ / __ __ / __ __ __ __ (dd-mmm-yyyy) |
| --- | --- |
| Site Number: | ________________ / _________________ |
| Research Assistant’s Name: | ___________________________________ |
| IDI number: | ___________________________________ |
| Type of participant (*choose the codes above*): | _________ |
| Start time (in 24 hour): | ________-Hours |
| End time (in 24 hour): | _______ - Hours |
| Duration of the interview: | ____: ____ (Minutes: Hours) |

**NB:** *Sections in* ***Italics*** *should not be read to participant. These are instructions to study staff.*

***Introduction***

1. **General**
2. Please tell me about your role in this facility
3. What are your primary duties?
4. **Now, I would like to ask you some general questions about the topic of our discussion:**
5. In your own words, what is Schistosomiasis?

*Probe: What do the people from this community call it?*

Do you know what female genital schistosomiasis is?

1. When a woman or girl has symptoms of schistosomiasis, where did they go to seek services?
2. What SRH services are offered in this community?

*Probe the kind of SRH services (referral, education etc)*

1. How are you involved in the delivery of SRH services?
2. What Schistosomiasis services are provided in within this facility? Community?

*Probe the kind of schistosomiasis services provided in both facility and community level*

1. How are you involved in the delivery of schistosomiasis services?
2. Who are the main clients who seek schistosomiasis services in this community?
3. Do you know how FGS is diagnosed?
4. **Acceptability of integrating FGS and SRH**
5. How would you integrate FGS and SRH services in community health services?
6. In your view, what do you think the results of integrating FGS and SRH services would be in your role?
   - *Probe for each reason provided*
7. How confident are you about providing the FGS-SRH services in your role?
   - *Please tell us why or why not.*
8. How acceptable do you think the integration would be to you as a provider?
   - *Probe each point shared*

5. How acceptable do you think the integration would be for the community?

- - *Probe each point shared*

1. **Feasibility of integrating FGS and SRH**
2. What kind of support would you require to enable you to integrate FGS and SRH services?
   - Probe:
     1. *Equipment*
     2. *Training*
     3. *Registers*
     4. *IEC Materials*
3. Based on your experience in this community, what are the potential barriers for integrating FGS and SRH services?

*Probe: Workload*

1. What would the enablers for integrating FGS and SRH services be in your roles in community health services?

Thank you so much for your time today. Do you have any additional thoughts or questions you would like to share with me?

**End Time**: ______ Hours

# IN-DEPTH INTERVIEW (IDI) GUIDE FOR COMMUNITY GATE KEEPERS

| Date of IDI: | __ __ / __ __ / __ __ __ __ (dd-mmm-yyyy) |
| --- | --- |
| Site Number: | ________________ / _________________ |
| Research Assistant’s Name | ___________________________________ |
| IDI number: | ___________________________________ |
| Language during interview *(NB: ensure consistency with consenting language)* | ___________________________________ |
| Start time (in 24 hour): | ________-Hours |
| End time (in 24 hour): | _______ - Hours |
| Duration of the interview: | ____: ____ (Minutes: Hours) |

**NB:** *Sections in* ***Italics*** *should not be read to participant. These are instructions to study staff.*

***Reminder:*** *After each question is asked, participants should be given plenty of time to respond. Be comfortable with silence, do not fill the silence with probes. Probes should be used to gather more information about the participant’s response, especially in the case of quiet participants. Probes are not required to be read. Probes should only be used when needed. Do not use more than one probe at once.*

1. **General understanding of Schistosomiasis**
2. Please tell me what you know about schistosomiasis? How is it called?
3. How does this community perceive schistosomiasis? Why?
4. How is schistosomiasis transmitted from one person to another?
5. What parts of the human body are affected by schistosomiasis?
6. How do you understand Female Genital Schistosomiasis?
7. What are the causes of Female Genital Schistosomiasis?
8. **Integration of FGS and SRH**

1. How would this community perceive SRH services with additional information on FGS?
   - *Probe: Community level, Health Facility level*
2. What is the implication of this integration for women/girls in your community?
   - *Probe for positive and negative and reasons for each*
3. Do you feel this integration will be maintained in your community within the SRH services? *probe: If yes, reasons? If No, reasons?*
4. Overall, how accepted do you think this integration will be in your community?
   - *Probe for reasons for acceptance at community level*
   - *Probe for whether they will understand it*
   - *Probe for the need to have it?*

1. Currently, when a woman or girl has symptoms of Schistosomiasis where do they go to seek for treatment services in this community?
   - *Probe where*
2. What kind of support is required to enable CHWs and health workers to integrate FGS into SRH?
3. Based on your experience in this community, what are the potential barriers for integrating FGS and SRH services?
   - *Probe for community level*
   - *Probe for facility level*
4. What are the potential enablers for integrating FGS and SRH?
   - *Probe for community level*
   - *Probe for facility level*
5. **Availability of health services (materials/equipment and healthcare workforce) to screen, diagnose and treat FGS during the integration**
6. What information do you think community health promoters/community health assistants share with community members during health education on female genital schistosomiasis?
7. How do you feel about the competency of CHP/CHAs in offering health education at community level?

**THE END**

We have come to the end of our discussion.

Do you have any comment regarding the topic we have discussed that you would want to share with us? Or, any question you would like to ask

Thank you so much for your time and responses.

**End Time**: ______ Hours

# IN-DEPTH INTERVIEW (IDI) GUIDE FOR WOMEN/GIRLS

| *Select participant type* | |  |
| --- | --- | --- |
| *Girls 15 – 18 years (1)* | *Women/girls 19-24 years (2)* | *Women/girls 25-49 years (3)* |

| Date of IDI: | __ __ / __ __ / __ __ __ __ (dd-mmm-yyyy) |
| --- | --- |
| Site Number: | ________________ / _________________ |
| Research Assistant’s Name: | ___________________________________ |
| Participant Type (*select code above*): | _________________ |
| IDI number: | ___________________________________ |
| Language during interview: *(NB: ensure consistency with consenting language)* | ___________________________________ |
| Start time (in 24 hour): | ________-Hours |
| End time (in 24 hour): | _______ - Hours |
| Duration of the interview: | ____: ____ (Minutes: Hours) |

**NB:** *Sections in* ***Italics*** *should not be read to participant. These are instructions to study staff.*

***Reminder:*** *After each question is asked, participants should be given plenty of time to respond. Be comfortable with silence, do not fill the silence with probes. Probes should be used to gather more information about the participant’s response, especially in the case of quiet participants. Probes are not required to be read. Probes should only be used when needed. Do not use more than one probe at once.*

1. **General understanding of Schistosomiasis and FGS**
2. Please tell me what you know about schistosomiasis/bilharzia? How is it called?
3. How does this community think about schistosomiasis/bilharzia? Why?
4. How can one get schistosomiasis/bilharzia?
5. What parts of the human body are affected by schistosomiasis/bilarzia?
6. Have you ever heard of Female Genital Schistosomiasis/bilharzia is?
7. What are the causes of Female Genital Schistosomiasis/bilharzia?
8. **Integration of FGS and Sexual Reproductive Health services**

1. How comfortable do you think you would be with the integration of FGS services into SRH services? *Probe for each reason provided*
2. Overall, do you think you would accept this integration?
   - *Probe further for each reason given*
3. Now, where do you go for treatment whenever you experience symptoms of schistosomiasis/bilharzia?
   - *Probe where*
4. What kind of support do you think you will require during this integration?

*Probe for information materials e g IECs etc?*

1. What do you think will be the challenges for receiving FGS and Sexual Reproductive Health services together?
   - *Probe for community level*
   - *Probe for facility level*
   - *Probe for individual level*
2. What do you think will help with receiving FGS and SRH services together?
   - *Probe for community level*
   - *Probe for facility level*
   - *Probe for individual level*
3. **Availability of health services (materials/equipment and healthcare workforce) to screen, diagnose and treat FGS during the integration**
4. When you came to this facility today, what information did the healthcare provider share with you about why you should be screened for female genital schistosomiasis/bilharzia?
5. How did you feel about the ability of the health care workers in offering the following services:

- Screening and checking for FGS?
- Diagnosing FGS?
- Treating FGS with medication

*Probe for each response provided above*

1. How did you feel about receiving female genital schistosomiasis services?
   - *Probe for community level*
   - *Probe for facility level*

**THE END**

We have come to the end of our discussion.

Do you have any comments regarding what we have discussed that you would want to share with us? Or, any questions you would like to ask?

Thank you so much for your time and responses.

**End Time**: ______ Hours

# FOCUS GROUP DISCUSSION (FGD) GUIDE FOR CHPs/CHAs/PEs

| *Has the participant been consented? Yes  (if no, do not proceed with the interview until consent is granted first)* | | |
| --- | --- | --- |
| *Select group of participants by age* | | |
| *CHP (1)* | *CHA (2)* | *Peer Educators (3)* |

| Date of FGD: | __ __ / __ __ / __ __ __ __ (dd-mmm-yyyy) |
| --- | --- |
| Site Number: | ________________ / _________________ |
| Research Assistant’s Name: | ___________________________________ |
| FGD number: | ___________________________________ |
| Type of participant (*choose the codes above*): | _________ |
| Start time (in 24 hour): | ________-Hours |
| End time (in 24 hour): | _______ - Hours |
| Duration of the interview: | ____: ____ (Minutes: Hours) |
| Total number of participants | ___ Females ______ Males ____total |

**NB:** *Sections in* ***Italics*** *should not be read to participant. These are instructions to study staff.*

***Introduction***

My name is _____________________from LVCT health. I will be your moderator for this discussion. My colleague is called __________________, s/he will be helping in taking notes during this discussion. The discussion will last for about 30 – 45 minutes. We thank you for agreeing to join this study. Female genital schistosomiasis (FGS) is a manifestation schistosomiasis (bilharzia) that affects the urinary and genital tract of the women/girls. I would like to remind you that the purpose of this discussion is to gain insight to better understand how acceptable and feasible it is to integrate female genital schistosomiasis (FGS) into sexual reproductive health (SRH) services. This will help us understand the factors that may increase the uptake and barriers that may have influenced the adoption, implementation, and sustainability of integrating FGS into SRH services. All your answers will be anonymous, and you can choose to not answer any question or stop participating at any time. We ask you, as a participant, to agree to keep information about others participating and the information discussed in the focus group confidential. For research purposes, I would like to record the discussion, is this okay for you? Could you please state that you agree to be recorded?

***Reminder:*** *After each question is asked, participants should be given plenty of time to respond. Be comfortable with silence, do not fill the silence with probes. Probes should be used to gather more information about the participant’s response, especially in the case of quiet participants. Probes are not required to be read. Probes should only be used when needed. Do not use more than one probe at once.*

1. **Acceptability of integrating FGS and SRH services**
2. How do you think the community would understand t the integration of FGS and SRH services?
3. In your view, would the integration of FGS and SRH services bring positive or negative impacts to the community?
   - *Probe for each reason provided*
4. How confident are you in providing the FGS-SRH services in your role?
   - *Please tell us why or why not.*
5. What changes in your role are needed ensure continuity of integration of FGS and SRH services?
   - *Probe each point shared*
6. Overall, how acceptable do you think it will be to integrate FGS and SRH services in your role?
   - *Probe the reasons*
7. **Feasibility of integrating FGS and SRH**
8. What kind of support would you require to enable you fit in with the healthcare workers during this integration?
   - Probe:
     1. *Training*
     2. *IEC materials*
9. How will you make community members realize the importance of this integration and screening for FGS?
10. Based on your experience in this community, what could potential challenges be in integrating FGS sn SRH services?

*Probe: Workload*

1. What do you think would support this integration?

We have come to the end of this discussion. Thank you so much for your time today. Do you have any additional thoughts or questions you would like to share with me?

**End Time**: ______ Hours

# FOCUS GROUP DISCUSSION (FGD) GUIDE FOR WOMEN/GIRLS

| *Has the participant been consented? Yes  (if no, do not proceed with the interview until consent is granted first)* | | |
| --- | --- | --- |
| *Select group of participants by age* | | |
| *Girls 15 – 18 years (1)* | *Women/Girls 19-24 years (2)* | *Women/Girls 25- 49 years (3)* |

| Date of FGD: | __ __ / __ __ / __ __ __ __ (dd-mmm-yyyy) |
| --- | --- |
| Site Number: | ________________ / _________________ |
| Moderator’s Name: | ___________________________________ |
| Note Taker’s Name: | ___________________________________ |
| Participant Type (*select code above*) | _________________ |
| Language during the discussion | ___________________________________ |
| Start time (in 24 hour): | ________-Hours |
| End time (in 24 hour): | _______ - Hours |
| Duration of the interview: | ____: ____ (Minutes: Hours) |
| \| Total number of participants \| \| --- \| | \| _____ \| \| --- \| |

**NB:** *Sections in* ***Italics*** *should not be read to participant. These are instructions to study staff.*

***Introduction***

My name is _____________________from LVCT health. I will be your moderator for this discussion. My colleague is called __________________, s/he will be helping in taking notes during this discussion. The discussion will last for about 30-45 minutes. Once again, we thank you for agreeing to join this study. Female genital schistosomiasis (FGS) is a manifestation of schistosomiasis (bilharzia) that affects the urinary and genital tract of women/girls. The purpose of this discussion is to better understand how the integration of female genital schistosomiasis (FGS) into sexual reproductive health (SRH) services will be accepted in this community. This will help us understand the factors that may increase or decrease the use of services by women and girls in this community. All your answers will be anonymous, your names will not be used and you can choose to not answer any question or stop participating at any time. We ask you, as a participant, to agree to keep information about others participating in this group confidential. For research purposes I would like to record the discussion, is this okay for you? Could you please state that you agree to be recorded?

**Discussion Rules**:

- Are there any rules you would like us to have during this discussion?
- You will be assigned numbers which will be used throughout the discussion. No one will be referred to by name. This helps to protect your privacy and makes it easier for the note taker to capture what has been said.
- Each person will be given time to speak. When one is speaking, others will be listening.
- Respecting the opinions/views of others. We may disagree with others’ arguments, but no one should tease the other.
- Everything discussed here should be kept confidential and should not be reported to anyone else.
- If you feel uncomfortable with anything during the discussion, you can raise your hand up and indicate that something is wrong. If you feel like dropping the discussion, that is fine.
- There is no right or wrong answers.

***Reminder:*** *After each question is asked, participants should be given plenty of time to respond. Be comfortable with silence, do not fill the silence with probes. Probes should be used to gather more information about the participant’s response, especially in the case of quiet participants. Probes are not required to be read. Probes should only be used when needed. Do not use more than one probe at once.*

**A. Experiences with integration of FGS and SRH**

1. Where do you currently receive the Sexual Reproductive Health and FGS services?
   - *Probe: where they received the services; Community level or Health Facility level and their experiences*
2. How do you feel about the services you receive about FGS?
   - *Probe for community level information*
   - *Probe for facility level information*
3. Do you think you will be satisfied with the SRH and FGS integration services?
4. Do you think this will help women in this community?
   - *Probe for each reason provided*
5. How comfortable are you with the idea of FGS services offered during SRH services? *Probe each reason/response provided*
6. Overall, how well will you accept the integration of FGS and SRH services?
   - *Probe for clarity for any reason provided*
7. When you have vaginal discharge, or pain during sexual intercourse or vaginal itchiness, what do you usually do? What kind of help do you look for? Where? With who? *Probe for each response provided to gain clarity*
8. With those symptoms, do you think you would know that you could have bilharzia (schsitosomiasis)?

*Probe if they know symptoms of schistosomiasis*

1. What kind of support would you need to receive from the healthcare providers when you visit the hospital with these symptoms?
2. Based on your experience, what do you think will make it hard to integrate FGS and SRH services?
   - *Probe for community level*
   - *Probe for facility level*
3. What do you think will make it possible to integrate these services?
   - *Probe for community level*
   - *Probe for facility level*
4. **Availability of health services (materials/equipment and healthcare workforce) to screen, diagnose and treat FGS during the intervention**
5. When you come to the facility, what information do the healthcare providers share with you about why you should be screened for female genital schistosomiasis?
6. How do you feel about the following services you receive from the healthcare workers at the facility during this intervention ?:

- Screening for FGS?
- Diagnosing or testing for FGS?
- Treating FGS with medication?

*Probe for each response provided above to check if they were happy with the services and satisfied*

**THE END**

We have come to the end of our discussion. Do you have any comments that you would want to share with us? Or, any questions you would like to ask

Thank you so much for your time and responses.

**End Time**: ______ Hours

# FOCUS GROUP DISCUSSION (FGD) GUIDE FOR COMMUNITY GATE KEEPERS

| *Has the participant been consented? Yes  (if no, do not proceed with the interview until consent is granted first)* |
| --- |

| Date of FGD: | __ __ / __ __ / __ __ __ __ (dd-mmm-yyyy) |
| --- | --- |
| Site Number: | ________________ / _________________ |
| Research Assistant’s Name: | ___________________________________ |
| FGD number: | ___________________________________ |
| Type of participant (*choose the codes above*): | _________ |
| Start time (in 24 hour): | ________-Hours |
| End time (in 24 hour): | _______ - Hours |
| Duration of the interview: | ____: ____ (Minutes: Hours) |
| Total number of participants | ___ Females ______ Males ____total |

**NB:** *Sections in* ***Italics*** *should not be read to participant. These are instructions to study staff.*

***Introduction***

My name is _____________________from LVCT health. I will be your moderator for this discussion. taking you through this discussion. My colleague is called __________________s/he will be helping in taking notes during this discussion. The discussion will last for about 30 – 45 minutes. Once again, we thank you for agreeing to join this study. Female genital schistosomiasis is a manifestation schistosomiasis (bilharzia) that affects the urinary and genital track of the women/girls. I would like to remind you that the purpose of this discussion is to gain insight to better understand how acceptable and feasible it is to integrate female genital schistosomiasis into sexual reproductive health services. This will help us understand the factors that may increase the uptake and barriers that may have influenced the adoption, implementation, and sustainability of integrating FGS into SRH services. All your answers will be anonymous, and you can choose to not answer or stop participating at any time. We ask you, as a participant, to agree to keep information about others participating and the information discussed in the focus group confidential. For research purposes I would like to record the discussion, is this okay for you? Could you please state that you agree to be recorded?

***Reminder:*** *After each question is asked, participants should be given plenty of time to respond. Be comfortable with silence, do not fill the silence with probes. Probes should be used to gather more information about the participant’s response, especially in the case of quiet participants. Probes are not required to be read. Probes should only be used when needed. Do not use more than one probe at once.*

1. **Acceptability of integrating FGS and SRH services**
2. How do you think the community will perceive the integration of FGS and SRH services?
3. In your view, what impacts do you think the integration of FGS and SRH services will bring to the community?
   - *Probe for each reason provided*
4. What do you think requires modification in to ensure continuity of integration of FGS and SRH services in this community?
   - *Probe each point shared*
5. Overall, how acceptable do you think it can be to integrate FGS and SRH services in this community?
   - *Probe the reasons*
6. **Feasibility of integrating FGS and SRH**
7. What kind of support would you require to enable you fit in this integration?
   - Probe:
     1. *Training*
     2. *IEC materials*
8. How will you make community members realize the importance of this integration?
9. Based on your experience in this community what can be the barriers during this integration?
10. What can be the enablers for this integration?

We have come to the end. Thank you so much for your time today. Do you have any additional thoughts or questions you would like to share with me?

**End Time**: ______ Hours

# KEY INFORMANT INTERVIEW (KII) GUIDE FOR: HEALTH MANAGERS

| *Has the participant been consented? Yes No. If no, ensure the participant is consented first.* | | |
| --- | --- | --- |
| ***Select Participant by type*** | | |
| *NTD coordinators (1)* | *Public Health Officers (2)* | *Health Coordinators (3)* |
| *Reproductive Health Coordinators (4)* | *Health Information officers (5)* | *Others, specify___________________* |

| Date of IDI: | __ __ / __ __ / __ __ __ __ (dd-mmm-yyyy) |
| --- | --- |
| Site Number: | ________________ / _________________ |
| Research Assistant’s Name: | ___________________________________ |
| Type of Participant (*Use code above* – *specify cadre*) | ______ -________ |
| IDI number: | _______________________________ |
| Start time (in 24 hour): | ________-Hours |
| End time (in 24 hour): | _______ - Hours |
| Duration of the interview: | ____: ____ (Minutes: Hours) |

**NB:** *Sections in* ***Italics*** *should not be read to participant. These are instructions to study staff.*

***Reminder:*** *After each question is asked, participants should be given plenty of time to respond. Be comfortable with silence, do not fill the silence with probes. Probes should be used to gather more information about the participant’s response, especially in the case of quiet participants. Probes are not required to be read. Probes should only be used when needed. Do not use more than one probe at once.*

1. ***Introduction***
2. Please tell me about your designation?
3. What are your primary duties?

Now, I would like to ask you some general questions about the topic of our discussion:

1. In your own words, what is Schistosomiasis?

1. What is female genital schistosomiasis?
2. What SRH services are offered within the facilities you support?

*Probe the kind of SRH services (diagnosis, counselling, treatment)*

1. Where are these SRH services offered?
2. How are you involved in the delivery of SRH services?
3. What Schistosomiasis services are provided in these facilities?

*Probe the kind of schistosomiasis services (diagnosis, counselling, treatment)*

1. How are you involved in the delivery of schistosomiasis services?
2. Who are the main healthcare providers involved in delivery of Schistosomiasis services?
3. What form of trainings for female genital schistosomiasis (FGS) have they received?
4. What services for addressing FGS are provided within these facilities?
5. **Acceptability of integrating FGS and SRH**
6. In your role, how would you integrate FGS and SRH services in these facilities?
7. In your view, what would be the implications of integrating FGS and SRH services?
   - *Probe for each reason provided*
8. In your role, how confident are you about providing integrated FGS and SRH services?
   - *Please tell us why or why not.*
9. What do you think will be required to be modified so that FGS and SRH services can be integrated?
   - *Probe each point shared*
10. Overall, how acceptable do you think it will be to integrate FGS and SRH services in these facilities?
    - *Probe the reasons*
11. **Feasibility of integrating FGS and SRH**
12. What kind of support would be required for FGS and SRH to be integrated in these facilities?
    - Probe:
      1. *Service delivery*
      2. *Equipment*
      3. *Human resources*
      4. *Finance*
      5. *Health information/Registers*
13. Based on your experience, what are the potential barriers for integrating FGS and SRH services in these facilities?
    - *Probe: Workload*
14. What would be the enablers for integrating FGS and SRH in these facilities?

Thank you so much for your time today. Do you have any additional thoughts or questions you would like to share with me?

**End Time**: ______ Hours

# Survey tool for acceptability of integrating FGS in SRH based on the Theoretical framework for Acceptability for female clients (15-49 years)

| Acceptability construct | TFA questionnaire items |
| --- | --- |
| **Affective attitude**  *How an individual feels about the intervention* | How comfortable did you feel being screened for FGS in the ANC/FP clinic?   \| Very uncomfortable \| Uncomfortable \| No opinion \| Comfortable \| Very comfortable \| \| --- \| --- \| --- \| --- \| --- \| \| 1 \| **2** \| **3** \| **4** \| **5** \| |
| **Burden**  *The amount of effort required to participate in the intervention* | How much extra time did it take to receive both FGS and SRH services?   \| No extra time at all \| A little extra time \| No opinion \| A lot of extra \| Huge amount of extra time \| \| --- \| --- \| --- \| --- \| --- \| \| 1 \| **2** \| **3** \| **4** \| **5** \| |
| **Ethicality**  *The extent to which the intervention has good fit with an individual’s value system* | How fair was integrating FGS in SRH services for women and girls?   \| Very unfair \| Unfair \| No opinion \| Fair \| Very fair \| \| --- \| --- \| --- \| --- \| --- \| \| 1 \| **2** \| **3** \| **4** \| **5** \| |
| **Perceived effectiveness**  *The extent to which the intervention is perceived to have achieved its objective* | The integration of FGS into SRH has improved the quality of your life?   \| Strongly disagree \| Disagree \| No opinion \| Agree \| Strongly agree \| \| --- \| --- \| --- \| --- \| --- \| \| 1 \| **2** \| **3** \| **4** \| **5** \| |
| **Intervention coherence**  *The extent to which the participant understands how the intervention works* | It was clear to me how integrating FGS in SRH would reduce my vulnerability to HIV infection   \| Strongly disagree \| Disagree \| No opinion \| Agree \| Strongly agree \| \| --- \| --- \| --- \| --- \| --- \| \| 1 \| **2** \| **3** \| **4** \| **5** \| |
| **Self -efficacy**  *A participant’s confidence that they can perform behavior (s) required to participate in the intervention* | How confident did you feel about receiving FGS in ANC/FP clinics?   \| Very unconfident \| Unconfident \| No opinion \| Confident \| Very confident \| \| --- \| --- \| --- \| --- \| --- \| \| 1 \| **2** \| **3** \| **4** \| **5** \| |
| **Opportunity costs**  *The benefits, profits or values that would have to be given up to engage with the intervention* | Integrating FGS services in SRH has interfered with the way you seek services   \| Strongly disagree \| Disagree \| No opinion \| Agree \| Strongly agree \| \| --- \| --- \| --- \| --- \| --- \| \| 1 \| **2** \| **3** \| **4** \| **5** \| |
| **General acceptability** | How acceptable was receiving integrated GS in SRH services to you?   \| Completely unacceptable \| Unacceptable \| No opinion \| Acceptable \| Completely acceptable \| \| --- \| --- \| --- \| --- \| --- \| \| 1 \| **2** \| **3** \| **4** \| **5** \| |

….………………………………………END………………………………………………………

# Survey tool for acceptability of integrating FGS in SRH based on the Theoretical framework for Acceptability for Health Workers

| Acceptability construct | TFA questionnaire items |
| --- | --- |
| **Affective attitude**  *How an individual feels about the intervention* | How comfortable did you feel integrating FGS in SRH services?   \| Very uncomfortable \| Uncomfortable \| No opinion \| Comfortable \| Very comfortable \| \| --- \| --- \| --- \| --- \| --- \| \| 1 \| **2** \| **3** \| **4** \| **5** \| |
| **Burden**  *The amount of effort required to participate in the intervention* | How much effort did it take to provide FGS services in SRH services?   \| No effort at all \| A little effort \| No opinion \| A lot of effort \| Huge effort \| \| --- \| --- \| --- \| --- \| --- \| \| 1 \| **2** \| **3** \| **4** \| **5** \| |
| **Ethicality**  *The extent to which the intervention has good fit with an individual’s value system* | How fair was integrating FGS in SRH services for women and girls?   \| Very unfair \| Unfair \| No opinion \| Fair \| Very fair \| \| --- \| --- \| --- \| --- \| --- \| \| 1 \| **2** \| **3** \| **4** \| **5** \| |
| **Perceived effectiveness**  *The extent to which the intervention is perceived to have achieved its objective* | The integration of FGS in SRH has improved the quality of live for women and girls   \| Strongly disagree \| Disagree \| No opinion \| Agree \| Strongly agree \| \| --- \| --- \| --- \| --- \| --- \| \| 1 \| **2** \| **3** \| **4** \| **5** \| |
| **Intervention coherence**  *The extent to which the participant understands how the intervention works* | It was clear to me how integrating FGS in SRH would reduce the vulnerability of women and girls to HIV infection   \| Strongly disagree \| Disagree \| No opinion \| Agree \| Strongly agree \| \| --- \| --- \| --- \| --- \| --- \| \| 1 \| **2** \| **3** \| **4** \| **5** \| |
| **Self -efficacy**  *A participant’s confidence that they can perform behavior (s) required to participate in the intervention* | How confident did you feel about integrating FGS in SRH services?   \| Very unconfident \| Unconfident \| No opinion \| Confident \| Very confident \| \| --- \| --- \| --- \| --- \| --- \| \| 1 \| **2** \| **3** \| **4** \| **5** \| |
| **Opportunity costs**  *The benefits, profits or values that would have to be given up to engage with the intervention* | Integrating FGS services in SRH has interfered with the way I work   \| Strongly disagree \| Disagree \| No opinion \| Agree \| Strongly agree \| \| --- \| --- \| --- \| --- \| --- \| \| 1 \| **2** \| **3** \| **4** \| **5** \| |
| **General acceptability** | How acceptable is integrating FGS in SRH services to you?   \| Completely unacceptable \| Unacceptable \| No opinion \| Acceptable \| Completely acceptable \| \| --- \| --- \| --- \| --- \| --- \| \| 1 \| **2** \| **3** \| **4** \| **5** \| |

….………………………………………END………………………………………………………
