# Supplementary material for: Acceptability and feasibility of integrating female genital schistosomiasis and sexual and reproductive health interventions in Kenya: A demonstration study
Source: PLOS Glob Public Health. 2025 Sep 17;5(9):e0004938. doi: 10.1371/journal.pgph.0004938 (PMC12443286; doi:10.1371/journal.pgph.0004938)
Supplement: S1 Table — (DOCX) [file pgph.0004938.s003.docx]

**Supplementary Information 3**

**Code Book**

| **Code** | **Description** | **Sub-code** |
| --- | --- | --- |
| **Health managers' perception** of Integrated FGS-SRH | Perceptions of managers who oversee the integration of FGS-SRH *(national, county, and sub-county level managers)* | **Sub-code 1.1:** Health manager perceptions on facilitators for integrating FGS and SRH  **Sub-code 1.2:** Health manager perceptions on challenges for integrating FGS and SRH |
| **Community Leaders Perception** of Integrated FGS-SRH | Perceptions of community leaders who shape opinions about the integration of FGS-SRH *(CHC members, village elders)* | **Sub-code 2.1:** Community leaders’ perceptions on facilitators for integrating FGS and SRH  **Sub-code 2.2:** Community leaders' perceptions on challenges for integrating FGS and SRH |
| **Client experience** of integrated FGs-SRH services | First-hand experience of women who received integrated FGS-SRH services | **Sub-code 3.1:** Client experience - FGS literacy  **Sub-code 3.2:** Client experience – Screening &Diagnosis  **Sub-code 3.3:** Client experience- Treatment  **Sub-code 3.4:** Client experience- Social Exclusion and Equity  **Sub-code 3.5:** Challenges experienced when accessing FGS-SRH services |
| **Health worker experience** of integrated FGs-SRH services | First-hand experience of health workers who deliver integrated FGS-SRH services *(Nurses, M.O, C.O, CHP, PHO)* | **Sub-code 4.1:** Health worker experience - FGS literacy  **Sub-code 4.2:** Health worker experience – Screening &Diagnosis  **Sub-code 4.3:** Health worker experience - Treatment  **Sub-code 4.4:** Health Worker Experience - Social Exclusion and Equity  **Sub-code 4.5:** Challenges when delivering FGS-SRH services |
| Client acceptability of integrated FGS-SRH services | We explored if clients accepted the integrated services | **Sub-code 5.1:** Clients - Overall acceptability of integrating FGS and SRH  **Sub-code 5.2:** Preferred place for receiving FGS-SRH services |
| Health Worker - Acceptability of integrated FGS and SRH services | We explored if health workers accepted the integrated services | **Sub-Code 6.1:** Preferred places for providing FGS-SRH services  **Sub-Code 6.2:** Outcomes of integrating FGS and SRH  **Sub-Code 6.3:** Confidence in integrating FGS and SRH  **Sub-Code 6.4:** Overall acceptability of integrating FGS and SRH |
| Feasibility of integrating FGS and SRH | We explored with health workers and managers on what will be required to integrate FGS-SRH successfully | **Sub-Code 7.1:** Investments required to integrate FGS and SRH |
