# Supplementary material for: Acceptability and feasibility of integrating female genital schistosomiasis and sexual and reproductive health interventions in Kenya: A demonstration study
Source: PLOS Glob Public Health. 2025 Sep 17;5(9):e0004938. doi: 10.1371/journal.pgph.0004938 (PMC12443286; doi:10.1371/journal.pgph.0004938)
Supplement: Attachment — Submitted filename: Rebuttal Letter to the Editor SIGNED.pdf [file pgph.0004938.s006.pdf]

Tuesday, 26 August 2025

The Editors in Chief,
PLOS Global Health

Re: Response to Reviewer Comments [PGPH-D-25-01741]

Many thanks for the review and feedback on the manuscript titled *"Acceptability and feasibility of integrating female genital schistosomiasis and sexual and reproductive health interventions in Kenya: a demonstration study"*. Please find the point-by-point response to each comment below:

Responses to review comments

Editor Comments

Editor comment # 1: Change "9" to "nine". All numbers under 10 should be spelled out in full. ***(Page 6 Line 124).***

Response to Editor comment # 1: We have changed the numbering as suggested.

Editor comment # 2: Consider removing these lines – while the additional context for why Sh transmission is high in these counties is interesting, it's not directly relevant to the paper (since it has already been established that surveys have shown high prevalence of urogenital schistosomiasis in these counties). Please keep the last sentence, related to the lack of data on the burden of FGS in these counties. ***(Page 5 and 6, Lines 121-131)***

Response to Editor comment # 2: We agree with this suggestion and deleted this text.

Editor comment # 3: Please be consistent with the use of decimals/number of significant figures listed – i.e., suggest changing 97% to 97.0% (if indeed that was the original result). ***(Page 11, Line 252).***

Response to Editor comment # 3: We have edited the decimals to improve consistency in the results section

Editor comment # 4: The "they" in this sentence is ambiguous – I believe it refers to the female patients (i.e., the female patients need permission from male partners to undergo a pelvic exam). Still, it could be misunderstood as referring to the men. Please adjust. ***(Page 12, Line 272).***

Response to Editor comment # 4: We have edited the text to improve the clarity

Editor comment # 5: Should this be a sub-heading, i.e., it falls under the broader umbrella of "Feasibility of integrating FGS and SRH among healthcare workers"? ***(Page 14, Line 335)***

Response to Editor comment # 5: We deleted this subheading and the subsequent one.to improve the flow of the findings on feasibility.

Off Argwings Kodhek Road P.O Box: 19835-00202, KNH Nairobi, GSM Lines: +254 724 256026, +254 722 203610, +254 733 333268.
Tel: +254 20 2646692, 2633212, Fax: +254 020 2633203.
Websites: www.lvcthealth.org; www.one2onekenya.org; www.lvctinstitute.ac.ke; www.gbvhivonline.com

Editor comment # 6: This seems to contradict the Methods, where it is stated that the three counties were selected due to the high prevalence of urogenital schistosomiasis. Or is the point here that within each county, there may be heterogeneity? Please clarify. ***(Page 23, Lines 541-543)***

> **Response to Editor comment # 6:** We have clarified that there is heterogeneity within the study counties.

<u>Reviewer # 1</u>

Reviewer feedback: This is a well-written manuscript. Limitations were clearly outlined, including limitations in conducting comparative or inferential statistical analyses. This study comes as a useful tool for assisting programme managers in integrating urogenital schistosomiasis into exiting SRH in endemic countries. The comments are intended to support advocacy efforts for the prioritization of urogenital schistosomiasis, a condition that remains significantly neglected in current health agendas

Reviewer comment # 1: I suggest rephrasing of this statement because it appears as if FGS can be completely treated with FGS, although part of the sentence indicates that praziquantel reduces parasitic burden. Furthermore, the findings of this study show that "a minor and an insignificant reduction in cervical lesions was observed in both of the two treatment arms" ***(Page 3 Line 60-61).*** The conclusion also states that "FGS-associated cervical lesions appear refractory to PZQ treatment even when this is administered in a repeated-dosing regimen".

> **Response to Reviewer comment # 1:** We have revised the sentence to indicate that administration of praziquantel reduces the worm burden. We did not have two treatment arms since this was an implementation study. We also did not report on the reduction of FGS lesions in the manuscript.

Reviewer comment # 2: Although this study focuses on integration of FGS with SRH, it may help to also mention integration of schistosomiasis vector control with the existing vector control strategies/programmes. ***(Page 3, Line 65-66)***

> **Response to Reviewer comment # 2:** We have expanded the sentence by adding vector control and added a reference for that addition.

Reviewer comment # 3: In some endemic countries, urogenital schistosomiasis is prevalent in some non-endemic areas due to importation from other counties or countries. It would be wise to mention if this is the case in Kenya and to indicate in the discussion if the findings and recommendations from this study would benefit communities in such settings. (***Page 6 Line 126 - 127).***

> **Response to Reviewer comment # 3:** This is a good suggestion. We would like to keep the narrative focused on the acceptability and feasibility of the integration in regions with high endemicity. We may receive additional funds to carry on this work and we'll incorporate this good proposal.

Reviewer comment # 4: Although the screening tool was used, it is not clear what was the criteria for referral. ***(Page 10, Line 234)***

> **Response to Reviewer comment # 4:** We have added a sentence in Table 2 that clients were referred for pelvic examination if they reported being in contact with stagnant freshwater and had at least one of the symptoms that are indicative of FGS.

Reviewer comment # 5: Maybe resources would not permit, but it would have been good to treat all these if they were referred due to findings that they were at risk. ***(Page 10, Line 237 and 238)***

Response to Reviewer comment # 5: It would have been ideal to treat all clients who were referred. However, some clients sought services elsewhere and others did not take up the referral immediately.

Reviewer comment # 6: Are these the only women who were offered praziquantel treatment or all who were screened and found to be at risk? ***(Page 10 Line 238)***

Response to Reviewer comment # 6: Yes. We have included a statement that the 2,301 clients who were diagnosed with FGS were treated with praziquantel

Reviewer comment # 7: It may be a good opportunity to mention integration with SRH in youth-friendly services for the youth and adolescents that are sexually active and have fresh water contact, to provide treatment for those at risk of urogenital schistosomiasis, even before FGS manifestation. ***(Page 21 line 501).***

Response to Reviewer comment # 7: That's a good idea. We have added this statement for advocacy "Additionally, integrating FGS services in youth friendly SRH clinics can provide health literacy, screening, diagnosis and treatment services to sexually active youth and adolescents who are in contact with stagnant water in endemic regions"

Reviewer comment # 8: It is not clear whether this integration will provide opportunities for prophylactic use of praziquantel to those who are at risk and have history of fresh water contact and do not have access to this treatment through school de-worming campaigns**. *(Page 21. line 507 and 508).***

Response to Reviewer comment # 8: This is an excellent thought. Our data does not speak to prophylactic chemotherapy. We can incorporate the prophylactic component in the next phase of the project. This paper wishes to limit its scope to the acceptability and feasibility of integrating the MSP in SRH services.

We have responded to each review comment in the resubmitted manuscript. We look forward to hearing from you regarding our re-submission.

Yours sincerely,

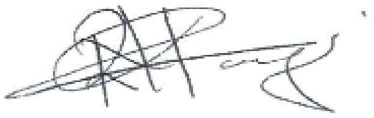

Dr. Robinson Karuga
On behalf of co-authors
